# A real-world data analysis of Ozanimod in the FDA Adverse Event Reporting System (FAERS) database

**DOI:** 10.1097/MD.0000000000044535

**Published:** 2025-09-12

**Authors:** Qinhui Tang, Xiaowei Tang, Xinyue Hu, Wenmeng Yin, Lian Luo, Yantong Li, Xiaolin Zhong

**Affiliations:** a Department of Gastroenterology, The Affiliated Hospital of Southwest Medical University, Luzhou, China; b Nuclear Medicine and Molecular Imaging Key Laboratory of Sichuan Province, Luzhou, China.

**Keywords:** adverse events, FAERS, Ozanimod, real-world data analysis

## Abstract

Ozanimod was approved in the United States in March 2020 for the treatment of relapsing multiple sclerosis and subsequently in 2021 for moderately to severely active ulcerative colitis. However, there is limited information available on the adverse drug events associated with its use. The main objective of this study was to explore the safety of Ozanimod after its market launch. Data was gathered from the United States Food and Drug Administration Adverse Event Reporting System database. To detect safety signals associated with Ozanimod adverse events, disproportionality analyses were performed using the reporting odds ratio, proportional reporting ratio, Bayesian confidence propagation neural network, and multi-item gamma Poisson shrinker algorithms. We extracted 7,118,789 reports from the FDA Adverse Event Reporting System database, of which 5429 reports identified Ozanimod as the primary suspect drug. Adverse reactions attributed to Ozanimod manifested across 26 organ systems, encompassing a total of 90 preferred terms meeting the criteria of all 4 algorithms simultaneously. In our study, back pain, hypertension, increased blood pressure, elevated hepatic enzymes, abnormal hepatic enzymes, pollakiuria, micturition urgency, and herpes zoster were consistent with the results of clinical trials. Notably, we observed some common adverse drug reaction signals, such as hypesthesia, muscle spasms, balance disorder, and gait disturbance, which were not documented in the official drug label. The majority of adverse events occurred within the initial 30 days following the initiation of Ozanimod treatment. Ozanimod presents the potential for diverse adverse reactions alongside its therapeutic benefits. Hence, in clinical practice, prompt detection of adverse drug reactions and the implementation of timely and effective preventive measures are essential.

## 1. Introduction

Ozanimod, a novel orally sphingosine-1-phosphate (S1P) receptor modulator, selectively targets S1P1 and S1P5 receptor subtypes to inhibit lymphocyte migration from lymphoid tissues, thereby reducing lymphocyte circulation and trafficking to inflammatory sites.^[1]^ This mechanism disrupts the pathological recruitment of autoreactive T cells and B cells to target organs, such as the central nervous system in multiple sclerosis (MS) or the colonic mucosa in ulcerative colitis (UC).^[2]^ Preclinical evidence suggests that modulating S1P1 and S1P5 receptors may have direct effects on the central nervous system, leading to a reduction in inflammatory cytokines, demyelination, axonal loss, and preservation of GABAergic transmission.^[3]^ Approved by the U.S. Food and Drug Administration (FDA) in March 2020 for relapsing MS and in May 2021 for moderate-to-severe UC, Ozanimod has become a cornerstone therapy for autoimmune diseases due to its dual anti-inflammatory and immunomodulatory effects.^[1,4]^ Its approval marked a milestone as the 1st S1P modulator indicated for both neurological and gastrointestinal autoimmune disorders, addressing critical unmet needs in patients with inadequate responses to conventional therapies.

Unlike 1st-generation S1P modulators such as fingolimod, Ozanimod high receptor selectivity minimizes binding to S1P3 receptors – a mechanism linked to cardiovascular adverse events (AEs) like bradycardia – potentially offering a safer therapeutic profile.^[2]^ This selectivity potentially offers a safer therapeutic profile, as evidenced by lower rates of symptomatic bradycardia in trials.^[5]^ Clinical trials demonstrated its efficacy in reducing relapse rates in MS and achieving clinical remission in UC. Common short-term AEs included herpes zoster infections, hypertension, and transient alanine aminotransferase elevations, with most events classified as mild to moderate.^[3,6]^ However, these trials predominantly enrolled younger, healthier populations with strict exclusion criteria for cardiovascular, hepatic, or renal comorbidities, potentially underestimating risks in real-world patients.

Post-marketing surveillance is crucial for identifying rare, delayed, or population-specific AEs that may not be detected in controlled trial settings. The FDA Adverse Event Reporting System (FAERS) database is the world’s largest publicly accessible repository of drug safety information, comprising a vast collection of AE reports submitted by healthcare professionals, pharmacists, manufacturers, and other individuals.^[7]^ To gain a more comprehensive understanding of the safety of Ozanimod in real-world clinical practice, we conducted signal mining and analysis of adverse drug events (ADEs) related to Ozanimod using the FAERS database. This study aimed to provide an in-depth understanding of the safety profile and AE characteristics of the drug in actual clinical use, offering guidance and recommendations for its clinical application.

## 2. Materials and methods

### 2.1. Data sources

FAERS is a database maintained by the U.S. FDA that collects and stores AE reports related to drugs and pharmaceutical products. The FAERS comprises 7 datasets, including patient demographic and administrative information (DEMO), drug information (DRUG), AEs (REAC), patient outcomes (OUTC), report sources (RPSR), therapy start states and end dates for reported drugs (THER), and indications for drug administration.^[8,9]^ The FAERS data were downloaded from the FAERS Quarterly Data Extract Files. Based on the date of FDA approval of Ozanimod, our study included all reports recorded in FAERS from the 1st quarter of 2020 to the 4th quarter of 2023.

### 2.2. Data extraction

**Figure 1.**
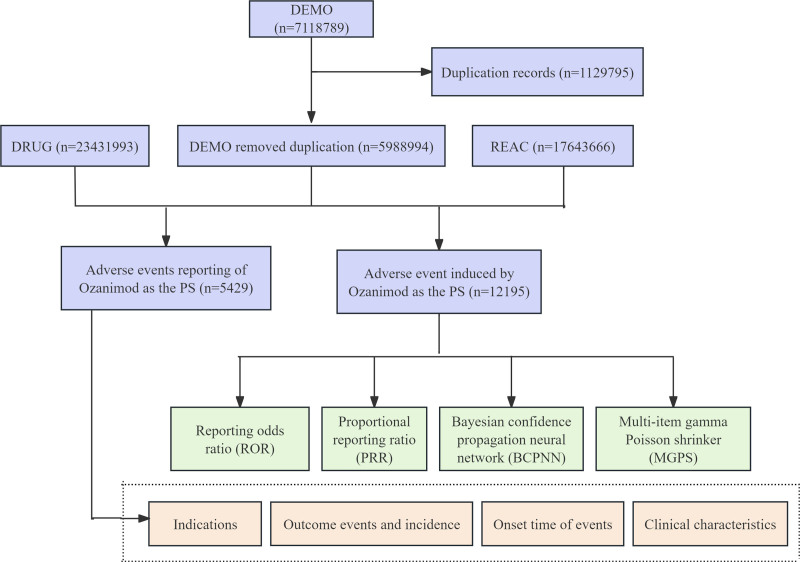
The flow diagram of selecting Ozanimod-related AEs from the FAERS database. AEs = adverse events, FAERS = FDA Adverse Event Reporting System, PS = primary suspected.

The drug’s role in AEs was coded using the following categories: primary suspect drug, secondary suspect drug, concomitant (C), and interacting (I). We defined the target drug as the generic name (Ozanimod) and the brand name (Zeposia), and selected the role_cod as primary suspect. Due to the spontaneous nature of reporting, duplicates are unavoidable. Therefore, we adhered to the FDA-recommended deduplication method: when the PRIMARYIDs were the same, we selected the latest FDA_DT; when both FDA_DT and CASEID were identical, we chose the higher PRIMARYID.^[10]^ The research workflow is illustrated in Figure 1. The AE names in the reports were standardized using the System Organ Classes (SOCs) and preferred terms (PTs) from the Medical Dictionary for Regulatory Activities.

### 2.3. Data mining algorithm

In our study, we employed disproportionality analysis, a method typically used in pharmacovigilance research, to generate potential signals between Ozanimod and adverse drug reactions. Four primary methodologies, including reporting odds ratio,^[11]^ proportional reporting ratio,^[12]^ Bayesian confidence propagation neural network,^[13]^ and multi-item gamma-Poisson shrinker models with empirical Bayesian geometric mean,^[14]^ were employed to assess the relationship between Ozanimod and adverse reactions. By employing this multi-method strategy, the aim is to overcome the limitations of a single algorithm, validate results from multiple perspectives, and detect more comprehensive and reliable safety signals, thereby enhancing the reliability and accuracy of data mining. In this study, the valid ADEs must simultaneously meet the positive signal criteria of all 4 aforementioned methods.^[15]^ The equations for the 4 algorithms and the criteria for positive signals are provided in Table S1, Supplemental Digital Content, https://links.lww.com/MD/P975. The period of onset is determined by the time lapse between EVENT_DT (the date of AE occurrence) and START_DT (the date of Ozanimod usage initiation). Moreover, any reports featuring input errors (where EVENT_DT precedes START_DT) or inaccuracies in date entries are disregarded.

## 3. Results

### 3.1. General characteristics

In this study, a total of 7118,789 AE reports were retrieved. After excluding duplicate entries, we obtained 5429 AE reports. Table 1 summarizes the basic characteristics of patients with Ozanimod. Reports related to Ozanimod were more common in female patients than in male patients, with percentages of 68.54% and 26.76%, respectively. Patients aged 18 to 64 years accounted for 65.52%, while elderly patients aged 65 years and older accounted for 10.37%. The reporting population was predominantly composed of health professionals (2626 cases, 48.37%), followed by consumers (1898 cases, 34.96%), and physicians (768 cases, 14.15%). From the perspective of report sources, the United States was the main country, accounting for as much as 92.69%. In clinical practice, reports leading to hospitalization or prolonged hospital stays, aside from some unspecified severe adverse reactions, were the most frequent, accounting for 7.85%. In 2020, due to the recent launch of the drug, there were relatively few reported AEs. The reported AEs remained relatively consistent across 2021, 2022, and 2023, with numbers consistently around 1800 each year.

**Table 1:** Characteristics of reports associated with Ozanimod.

	Ozanimod	Percentage
	Case number	
Number of events	5429	
Gender		
Male	1453	26.76%
Female	3721	68.54%
Unknown	255	4.70%
Age		
<18	2	0.04%
18 ≥ and<65	3557	65.52%
≥65	563	10.37%
Unknown	1307	24.07%
Reported countries		
United States	5032	92.69%
Germany	146	2.69%
Other country	251	4.62%
Reported person		
Physician	768	14.15%
Consumer	1898	34.96%
Pharmacist	121	2.23%
Health professional	2626	48.37%
Lawyer	2	0.04%
Unknown	14	0.26%
Serious outcomes		
Death	58	1.07%
Disability	51	0.94%
Life-threatening	50	0.92%
Hospitalization – initial or prolonged	426	7.85%
Other outcome	948	17.46%
Unknown	3896	71.76%
Reporting year		
2020	214	3.94%
2021	1956	36.03%
2022	1739	32.03%
2023	1520	28.00%

### 3.2. Signal detection

As shown in Table 2, it was found that the drug-induced adverse reactions in 26 SOCs. Among the SOCs evaluated, nervous system disorders was the only category that satisfied all 4 predefined criteria. Additionally, general disorders and administration site conditions, gastrointestinal disorders, investigations, musculoskeletal and connective tissue disorders, eye disorders, and vascular disorders were significant SOCs that met 2 out of the 4 criteria. The most frequently reported AEs by system organ class were general disorders and administration site conditions (n = 2342), followed by nervous system disorders (n = 2225) and gastrointestinal disorders (n = 1485).

**Table 2:** Signal strength of reports of Ozanimod at the System Organ Class (SOC) level in FAERS database.

System Organ Class (SOC)	Case reports	ROR (95% Cl)	PRR (χ2)	EBGM (EBGM05)	IC (IC025)
General disorders and administration site conditions	2342	1.09 (1.04–1.14)*	1.07 (14.46)	1.07 (1.03)	0.1 (0.04)*
Nervous system disorders	2225	2.86 (2.74–3)*	2.52 (2204.47)*	2.52 (2.43)*	1.33 (1.27)*
Gastrointestinal disorders	1485	1.67 (1.58–1.77)*	1.59 (352)	1.59 (1.52)	0.67 (0.59)*
Investigations	880	1.27 (1.19–1.36)*	1.25 (48.07)	1.25 (1.18)	0.33 (0.23)*
Musculoskeletal and connective tissue disorders	843	1.39 (1.29–1.49)*	1.36 (85.22)	1.36 (1.28)	0.44 (0.34)*
Infections and infestations	719	1.08 (1–1.16)	1.07 (3.74)	1.07 (1.01)	0.1 (−0.01)
Injury, poisoning, and procedural complications	668	0.42 (0.39–0.45)	0.45 (507.94)	0.45 (0.42)	−1.15 (−1.26)
Psychiatric disorders	532	0.76 (0.7–0.83)	0.77 (36.94)	0.77 (0.72)	−0.37 (−0.5)
Respiratory, thoracic, and mediastinal disorders	423	0.77 (0.7–0.85)	0.78 (27.66)	0.78 (0.72)	−0.36 (−0.5)
Skin and subcutaneous tissue disorders	377	0.55 (0.5–0.61)	0.57 (131.78)	0.57 (0.52)	−0.82 (−0.97)
Eye disorders	313	1.35 (1.21–1.51)*	1.34 (27.88)	1.34 (1.22)	0.42 (0.26)*
Vascular disorders	312	1.4 (1.25–1.56)*	1.39 (34.16)	1.39 (1.26)	0.47 (0.31)*
Cardiac disorders	197	0.82 (0.71–0.94)	0.82 (8.04)	0.82 (0.73)	−0.29 (−0.49)
Renal and urinary disorders	158	0.68 (0.58–0.79)	0.68 (23.61)	0.68 (0.6)	−0.55 (−0.78)
Blood and lymphatic system disorders	133	0.63 (0.53–0.75)	0.64 (27.76)	0.64 (0.55)	−0.65 (−0.9)
Metabolism and nutrition disorders	128	0.55 (0.46–0.66)	0.56 (46.08)	0.56 (0.48)	−0.84 (−1.1)
Neoplasms benign, malignant, and unspecified (incl cysts and polyps)	116	0.21 (0.17–0.25)	0.21 (349.57)	0.21 (0.18)	−2.22 (−2.49)
Immune system disorders	76	0.55 (0.44–0.69)	0.55 (27.55)	0.55 (0.46)	−0.85 (−1.18)
Reproductive system and breast disorders	59	0.82 (0.63–1.05)	0.82 (2.43)	0.82 (0.66)	−0.29 (−0.66)
Ear and labyrinth disorders	55	1.12 (0.86–1.47)	1.12 (0.76)	1.12 (0.9)	0.17 (−0.22)
Surgical and medical procedures	38	0.21 (0.15–0.29)	0.22 (110.36)	0.22 (0.16)	−2.22 (−2.68)
Hepatobiliary disorders	36	0.36 (0.26–0.5)	0.36 (40.3)	0.36 (0.28)	−1.46 (−1.93)
Pregnancy, puerperium, and perinatal conditions	36	0.86 (0.62–1.2)	0.86 (0.78)	0.86 (0.66)	−0.21 (−0.69)
Social circumstances	17	0.3 (0.18–0.48)	0.3 (28.54)	0.3 (0.2)	−1.75 (−2.43)
Product issues	15	0.06 (0.04–0.11)	0.07 (201.54)	0.07 (0.04)	−3.92 (−4.64)
Endocrine disorders	12	0.38 (0.22–0.68)	0.39 (11.79)	0.39 (0.24)	−1.38 (−2.18)

CI = confidence interval, EBGM = empirical Bayesian geometric mean, IC = information component, PRR = proportional reporting ratio, ROR = reporting odds ratio, SOC = System Organ Class, χ2 = chi-squared.

* Indicates statistically significant signals in algorithm.

After excluding nonmeaningful PTs, including AEs, unevaluable events, and labeled drug-drug interaction issues, the 4 algorithms concurrently identified 90 Ozanimod-associated AE signals across 26 SOCs. The number of reported PTs ≥ 35 is shown in Table 3, while the rest are listed in Table S2, Supplemental Digital Content, https://links.lww.com/MD/P975. The top 5 PTs ranked by the number of cases were fatigue (n = 518), headache (n = 393), dizziness (n = 270), back pain (n = 161), and hypoaesthesia (n = 118). According to previous studies on Ozanimod, infections, cardiac toxicity events, hepatic toxicity events, and ocular disorders were commonly reported. In our research, we found that back pain, hypertension, increased blood pressure, elevated hepatic enzymes, abnormal hepatic enzymes, pollakiuria, micturition urgency, and herpes zoster were consistent with the results of clinical trials. Unexpected AEs included PTs of hypesthesia, muscle spasms, balance disorder, gait disturbance, paraesthesia and so on.

**Table 3:** Signal strength of reports of Ozanimod at the preferred terms (PT) level in FAERS database.

PT	Case reports	ROR (95% Cl)	PRR (χ2)	EBGM (EBGM05)	IC (IC025)
Fatigue	518	3.48 (3.18–3.8)	3.37 (872.97)	3.37 (3.13)	1.75 (1.62)
Headache	393	3.65 (3.3–4.03)	3.56 (728.6)	3.55 (3.27)	1.83 (1.68)
Dizziness	270	3.28 (2.91–3.7)	3.23 (417.59)	3.22 (2.91)	1.69 (1.51)
Back pain	161	3.95 (3.38–4.62)	3.91 (349.07)	3.9 (3.43)	1.96 (1.74)
Hypoaesthesia	118	4.74 (3.95–5.68)	4.7 (343.49)	4.69 (4.03)	2.23 (1.96)
Hypertension	106	2.76 (2.28–3.34)	2.74 (117.26)	2.74 (2.33)	1.45 (1.17)
Depression	98	2.81 (2.3–3.42)	2.79 (112.79)	2.79 (2.36)	1.48 (1.19)
Muscle spasms	87	2.97 (2.41–3.67)	2.96 (113)	2.96 (2.48)	1.56 (1.25)
Balance disorder	86	5.86 (4.74–7.25)	5.82 (342.68)	5.8 (4.86)	2.54 (2.23)
Blood pressure increased	85	2.85 (2.3–3.53)	2.84 (101.04)	2.83 (2.37)	1.5 (1.19)
Gait disturbance	84	2.46 (1.98–3.05)	2.45 (72.15)	2.45 (2.04)	1.29 (0.98)
Paraesthesia	84	3.15 (2.54–3.9)	3.13 (121.87)	3.13 (2.61)	1.64 (1.33)
Memory impairment	77	2.97 (2.37–3.71)	2.95 (99.57)	2.95 (2.45)	1.56 (1.23)
Migraine	76	4.11 (3.28–5.15)	4.09 (176.98)	4.08 (3.38)	2.03 (1.7)
White blood cell count decreased	73	3.14 (2.49–3.95)	3.13 (105.61)	3.12 (2.58)	1.64 (1.31)
Visual impairment	72	2.8 (2.22–3.53)	2.79 (82.75)	2.79 (2.3)	1.48 (1.14)
Lymphocyte count decreased	72	17.11 (13.55–21.6)	17.01 (1072.83)	16.83 (13.84)	4.07 (3.73)
Hemorrhage	71	3.99 (3.16–5.04)	3.97 (157.59)	3.96 (3.26)	1.99 (1.64)
Hematochezia	67	5.59 (4.4–7.11)	5.57 (250.38)	5.55 (4.54)	2.47 (2.12)
Heart rate decreased	66	7.87 (6.17–10.02)	7.83 (391.26)	7.79 (6.36)	2.96 (2.61)
Palpitations	66	3.44 (2.7–4.38)	3.42 (113.12)	3.42 (2.79)	1.77 (1.42)
Vision blurred	64	2.88 (2.25–3.69)	2.87 (78.13)	2.87 (2.34)	1.52 (1.16)
Stress	60	4.17 (3.23–5.37)	4.15 (143.21)	4.14 (3.35)	2.05 (1.68)
Musculoskeletal stiffness	54	2.85 (2.18–3.73)	2.84 (64.54)	2.84 (2.27)	1.51 (1.12)
Lymphopenia	54	17.13 (13.09–22.41)	17.06 (806.9)	16.87 (13.47)	4.08 (3.68)
Hepatic enzyme increased	50	3.62 (2.74–4.78)	3.61 (94.13)	3.6 (2.85)	1.85 (1.44)
Muscular weakness	46	2.58 (1.93–3.44)	2.57 (44.08)	2.57 (2.01)	1.36 (0.94)
Rectal hemorrhage	40	5.89 (4.31–8.04)	5.87 (161.12)	5.85 (4.51)	2.55 (2.1)
Herpes zoster	35	2.97 (2.13–4.14)	2.97 (45.54)	2.96 (2.24)	1.57 (1.08)

CI = confidence interval, EBGM = empirical Bayesian geometric mean, IC = information component, PRR = proportional reporting ratio, PT = preferred term, ROR = reporting odds ratio, χ2 = chi-squared.

### 3.3. Onset time of events

**Figure 2.**
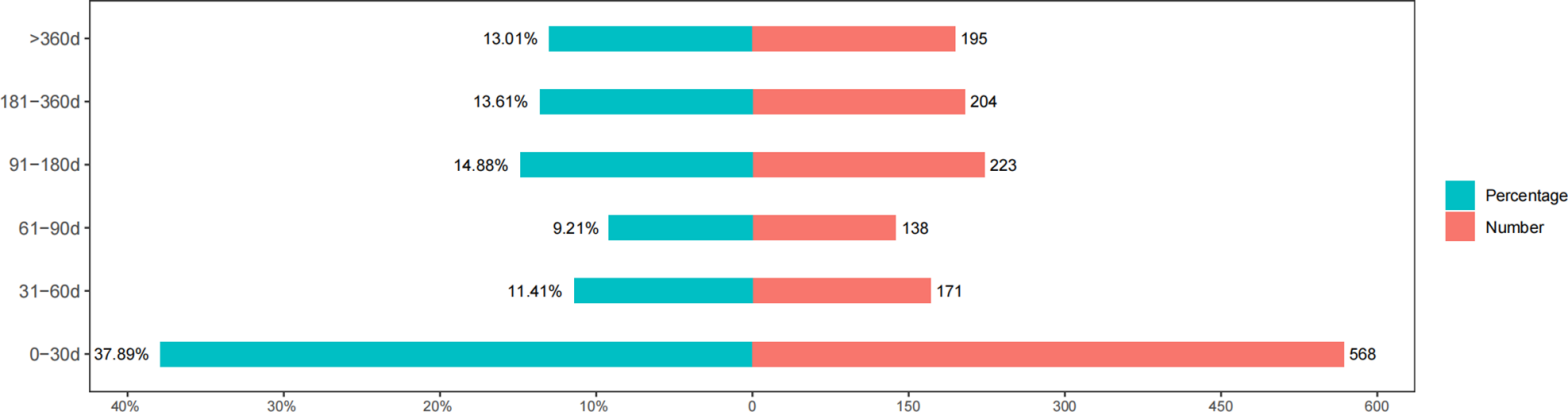
Time-to-onset of Ozanimod-related AEs. AEs = adverse events.

The onset times of Ozanimod-associated AEs were collected from the database. Excluding unreported or unknown onset time reports, a total of 1499 cases reported onset time. As shown in Figure 2, the onset of Ozanimod-related AEs may extend beyond 1 year (n = 195, 13.01%). Still, the majority of AEs occurred within 1 month after medication initiation (n = 568, 37.89%). There were also occurrences within 2 months (n = 171, 11.41%), 3 months (n = 138, 9.21%), 6 months (n = 223, 14.88%), and twelve months (n = 204, 13.61%). Therefore, early identification of adverse reactions caused by Ozanimod treatment can help doctors take timely and effective preventive measures, reducing the occurrence of adverse reactions in patients.

## 4. Discussion

Ozanimod, which acts by modulating S1P receptors, exhibits a distinct mechanism that influences both the immune system and vascular integrity. Clinical trials have highlighted its efficacy in reducing the relapse rate and radiological markers of disease activity in MS by preventing lymphocyte egress from lymph nodes, thereby reducing inflammatory activity within the central nervous system.^[16]^ A dose-blinded extension of a randomized phase II study indicates that Ozanimod demonstrates sustained efficacy in participants with MS treated for up to 2 years and achieves similar effects in participants who switched from placebo.^[17]^ Furthermore, Ozanimod was more effective than placebo as induction and maintenance therapy in patients with moderately to severely active UC in the randomized, double-blind, placebo-controlled, multinational phase II TOUCHSTONE trial.^[18]^

As the use of Ozanimod becomes increasingly widespread in the treatment of MS and UC, there is a growing concern about its safety. In previous studies, research on Ozanimod has primarily focused on its mechanisms, clinical trials, and literature analysis, with few articles addressing the latest real-world research. FAERS is a key tool for drug safety monitoring, providing valuable resources for researchers to identify new drug safety issues and trends. Using the FAERS database, this study summarized and assessed AE reports related to Ozanimod in the post-marketing setting.

The incidence of adverse reactions to Ozanimod was significantly higher in females (68.54%) compared to males (26.76%). This may be related to the increase in female patients with MS, leading to increased opportunities for drug use. Studies have shown that females are twice as likely to live with MS as males.^[19]^ Adverse reactions accounted for the majority in the age group of 18 to 64 years, which may be attributed to the fact that MS and inflammatory bowel disease predominantly occur in this age range.^[20,21]^ Although Ozanimod has demonstrated specific therapeutic efficacy in clinical settings, patients may still experience adverse reactions during treatment. Early identification of AEs is necessary, as these effects may be life-threatening or lead to disease progression.

According to the disproportionality analysis, the most significant signal at System Organ Class level was nervous system disorders. This finding aligns with the known pharmacological action of Ozanimod as a S1P receptor modulator, which affects lymphocyte trafficking and may lead to central and peripheral neurological manifestations.^[3]^ According to the prescribing information, Ozanimod may cause several adverse reactions, including infections, bradyarrhythmia, atrioventricular conduction delays, liver injury, fetal risk, increased blood pressure, respiratory effects, macular edema, posterior reversible encephalopathy syndrome, and so forth. In a pooled analysis of tolerability data from SUNBEAM and phase III RADIANCE, the most common adverse reactions in Ozanimod recipients were upper respiratory infection, hepatic transaminase elevation, orthostatic hypotension, urinary tract infection, back pain, hypertension, and upper abdominal pain. The most frequently reported AEs in our study, including fatigue, headache, and dizziness, were consistent with observations from pivotal Ozanimod trials.^[3,16]^ These symptoms are likely attributable to S1P receptor modulation, which can transiently affect vascular tone and autonomic function.^[22]^ Additionally, back pain, hypertension, increased hepatic enzymes, and herpes zoster infections, which were previously documented in clinical studies, were also detected in our analysis, validating the reliability of FAERS data in capturing known Ozanimod-associated risks.^[18]^ Notably, hepatic enzyme elevations and herpes zoster infections, both of which are recognized as class effects of S1P receptor modulators (such as fingolimod, siponimod), were prominent in our dataset. This suggests that Ozanimod shares similar safety considerations with other drugs in its class, particularly regarding liver function monitoring and vaccination strategies before initiation.^[23]^

Beyond the expected AEs, our study identified several unexpected signals, including hypesthesia, muscle spasms, balance disorders, and gait disturbances. These neurological and musculoskeletal events may reflect off-target S1P receptor effects in the central nervous system and peripheral nerves.^[24]^ Preclinical studies have demonstrated that S1P receptors are widely expressed in neural tissues, and their modulation may contribute to sensory and motor dysfunction.^[25]^ While these AEs were not major highlights in clinical trials, their detection in real-world settings underscores the importance of post-marketing surveillance in identifying rare or delayed-onset toxicities. At the same time, we should pay attention to adverse reactions such as abnormal lymphocyte count, abnormal magnetic resonance imaging head, traumatic fracture, and uterine disorder. However, their occurrence is relatively low, and their high signal intensity requires more attention and research.

Our research findings indicated that the median time to onset was 62 days, with the majority of cases occurring within the 1st month after starting treatment with Ozanimod (n = 568, 37.89%). The median onset times for fatigue, headaches, and dizziness were 29 days, 20.5 days, and 22 days, respectively. We must pay particular attention to AEs that may occur in patients during the initial month of treatment. Early identification of adverse reactions caused by Ozanimod therapy can help alleviate patient discomfort and potentially prevent life-threatening situations.

While this study provides valuable insights into Ozanimod real-world safety profile, the inherent limitations of the FAERS database – including information gaps, duplicate reports, inconsistent formatting, and ADE misclassification – must be acknowledged, as these factors may introduce analytical bias. Due to the possibility of incomplete reporting and reporting bias, our results need to be interpreted with caution. Additionally, the data in the FAERS database cannot directly establish causality, so we cannot rely solely on it to determine the causal relationship between Ozanimod and specific AEs. Multiple factors, including drug characteristics, individual differences, and underlying conditions, influence the occurrence of AEs. Moreover, since the data were predominantly derived from Western countries, this geographic limitation may introduce regional bias, potentially limiting the generalizability of the observed adverse drug reaction profiles to heterogeneous global populations.

## 5. Conclusion

Our pharmacovigilance analysis of real-world data in the FAERS database revealed safety signals and potential risks associated with the use of Ozanimod. The study revealed common adverse reactions not explicitly mentioned in the drug label, such as hypesthesia, muscle spasms, balance disorder, gait disturbance, and paraesthesia. It is worth noting that, despite the relatively low frequency of certain adverse reactions (such as abnormal lymphocyte count, abnormal magnetic resonance imaging head, and traumatic fracture), their high signal intensity necessitates more attention and research. However, to gain a more comprehensive and precise understanding, future studies may consider adopting stricter prospective research methodologies that combine clinical trials with epidemiological investigations. This approach would enable a more accurate evaluation of the safety risks associated with Ozanimod.

## Author contributions

**Data curation:** Xiaowei Tang.

**Software:** Xinyue Hu, Lian Luo, Yantong Li.

**Supervision:** Wenmeng Yin.

**Writing – original draft:** Qinhui Tang.

**Writing – review & editing:** Xiaolin Zhong.

## Supplementary Material


