# Supplementary material for: A real-world data analysis of Ozanimod in the FDA Adverse Event Reporting System (FAERS) database
Source: Medicine (Baltimore). 2025 Sep 12;104(37):e44535. doi: 10.1097/MD.0000000000044535 (PMC12440506; doi:10.1097/MD.0000000000044535)
Supplement: Supplementary file 1 [file medi-104-e44535-s001.docx]

Supplementary Table 1. Four major algorithms used for signal detection.

| Algorithms | Equation | Criteria |
| --- | --- | --- |
| ROR | ROR=ad/b/c | lower limit of 95% CI>1, N≥3 |
|  | 95%CI=e^ln(ROR)±1.96(1/a+1/b+1/c+1/d)^0.5^ |  |
| PRR | PRR=a(c+d)/c/(a+b) | PRR≥2, χ^2^≥4, N≥3 |
|  | χ^2^=[(ad-bc)^2](a+b+c+d)/[(a+b)(c+d)(a+c)(b+d)] |  |
| BCPNN | IC=log_2_a(a+b+c+d)(a+c)(a+b) | IC025>0 |
|  | 95%CI= E(IC) ± 2V(IC)^0.5 |  |
| MGPS | EBGM=a(a+b+c+d)/(a+c)/(a+b) | EBGM05>2 |
|  | 95%CI=e^ln(EBGM)±1.96(1/a+1/b+1/c+1/d)^0.5^ |  |

Equation: a, number of reports containing both the target drug and target adverse drug reaction; b, number of reports containing other adverse drug reaction of the target drug; c, number of reports containing the target adverse drug reaction of other drugs; d, number of reports containing other drugs and other adverse drug reactions. 95%CI, 95% confidence interval; *N*, the number of reports; χ^2^, chi-squared; IC, information component; IC025, the lower limit of 95% CI of the IC; E(IC), the IC expectations; V(IC), the variance of IC; EBGM, empirical Bayesian geometric mean; EBGM05, the lower limit of 95% CI of EBGM.

Supplementary Table 2. Signal strength of all reports associated with Ozanimod at the Preferred Term level in the FAERS database. PT, Preferred Term; ROR, Reporting Odds Ratio; CI, Confidence Interval; PRR, Proportional Reporting Ratio; χ2, Chi-squared; EBGM, Empirical Bayesian Geometric Mean; IC, Information Component.

| **PT** | Case Reports | ROR(95%Cl) | PRR(**χ2**) | EBGM(EBGM05) | IC(IC025) |
| --- | --- | --- | --- | --- | --- |
| Fatigue | 518 | 3.48 ( 3.18 - 3.8 ) | 3.37 ( 872.97 ) | 3.37 ( 3.13 ) | 1.75 ( 1.62 ) |
| Headache | 393 | 3.65 ( 3.3 - 4.03 ) | 3.56 ( 728.6 ) | 3.55 ( 3.27 ) | 1.83 ( 1.68 ) |
| Dizziness | 270 | 3.28 ( 2.91 - 3.7 ) | 3.23 ( 417.59 ) | 3.22 ( 2.91 ) | 1.69 ( 1.51 ) |
| Back pain | 161 | 3.95 ( 3.38 - 4.62 ) | 3.91 ( 349.07 ) | 3.9 ( 3.43 ) | 1.96 ( 1.74 ) |
| Hypoaesthesia | 118 | 4.74 ( 3.95 - 5.68 ) | 4.7 ( 343.49 ) | 4.69 ( 4.03 ) | 2.23 ( 1.96 ) |
| Hypertension | 106 | 2.76 ( 2.28 - 3.34 ) | 2.74 ( 117.26 ) | 2.74 ( 2.33 ) | 1.45 ( 1.17 ) |
| Depression | 98 | 2.81 ( 2.3 - 3.42 ) | 2.79 ( 112.79 ) | 2.79 ( 2.36 ) | 1.48 ( 1.19 ) |
| Muscle spasms | 87 | 2.97 ( 2.41 - 3.67 ) | 2.96 ( 113 ) | 2.96 ( 2.48 ) | 1.56 ( 1.25 ) |
| Balance disorder | 86 | 5.86 ( 4.74 - 7.25 ) | 5.82 ( 342.68 ) | 5.8 ( 4.86 ) | 2.54 ( 2.23 ) |
| Blood pressure increased | 85 | 2.85 ( 2.3 - 3.53 ) | 2.84 ( 101.04 ) | 2.83 ( 2.37 ) | 1.5 ( 1.19 ) |
| Gait disturbance | 84 | 2.46 ( 1.98 - 3.05 ) | 2.45 ( 72.15 ) | 2.45 ( 2.04 ) | 1.29 ( 0.98 ) |
| Paraesthesia | 84 | 3.15 ( 2.54 - 3.9 ) | 3.13 ( 121.87 ) | 3.13 ( 2.61 ) | 1.64 ( 1.33 ) |
| Memory impairment | 77 | 2.97 ( 2.37 - 3.71 ) | 2.95 ( 99.57 ) | 2.95 ( 2.45 ) | 1.56 ( 1.23 ) |
| Migraine | 76 | 4.11 ( 3.28 - 5.15 ) | 4.09 ( 176.98 ) | 4.08 ( 3.38 ) | 2.03 ( 1.7 ) |
| White blood cell count decreased | 73 | 3.14 ( 2.49 - 3.95 ) | 3.13 ( 105.61 ) | 3.12 ( 2.58 ) | 1.64 ( 1.31 ) |
| Visual impairment | 72 | 2.8 ( 2.22 - 3.53 ) | 2.79 ( 82.75 ) | 2.79 ( 2.3 ) | 1.48 ( 1.14 ) |
| Lymphocyte count decreased | 72 | 17.11 ( 13.55 - 21.6 ) | 17.01 ( 1072.83 ) | 16.83 ( 13.84 ) | 4.07 ( 3.73 ) |
| Haemorrhage | 71 | 3.99 ( 3.16 - 5.04 ) | 3.97 ( 157.59 ) | 3.96 ( 3.26 ) | 1.99 ( 1.64 ) |
| Haematochezia | 67 | 5.59 ( 4.4 - 7.11 ) | 5.57 ( 250.38 ) | 5.55 ( 4.54 ) | 2.47 ( 2.12 ) |
| Heart rate decreased | 66 | 7.87 ( 6.17 - 10.02 ) | 7.83 ( 391.26 ) | 7.79 ( 6.36 ) | 2.96 ( 2.61 ) |
| Palpitations | 66 | 3.44 ( 2.7 - 4.38 ) | 3.42 ( 113.12 ) | 3.42 ( 2.79 ) | 1.77 ( 1.42 ) |
| Vision blurred | 64 | 2.88 ( 2.25 - 3.69 ) | 2.87 ( 78.13 ) | 2.87 ( 2.34 ) | 1.52 ( 1.16 ) |
| Stress | 60 | 4.17 ( 3.23 - 5.37 ) | 4.15 ( 143.21 ) | 4.14 ( 3.35 ) | 2.05 ( 1.68 ) |
| Musculoskeletal stiffness | 54 | 2.85 ( 2.18 - 3.73 ) | 2.84 ( 64.54 ) | 2.84 ( 2.27 ) | 1.51 ( 1.12 ) |
| Lymphopenia | 54 | 17.13 ( 13.09 - 22.41 ) | 17.06 ( 806.9 ) | 16.87 ( 13.47 ) | 4.08 ( 3.68 ) |
| Hepatic enzyme increased | 50 | 3.62 ( 2.74 - 4.78 ) | 3.61 ( 94.13 ) | 3.6 ( 2.85 ) | 1.85 ( 1.44 ) |
| Muscular weakness | 46 | 2.58 ( 1.93 - 3.44 ) | 2.57 ( 44.08 ) | 2.57 ( 2.01 ) | 1.36 ( 0.94 ) |
| Rectal haemorrhage | 40 | 5.89 ( 4.31 - 8.04 ) | 5.87 ( 161.12 ) | 5.85 ( 4.51 ) | 2.55 ( 2.1 ) |
| Herpes zoster | 35 | 2.97 ( 2.13 - 4.14 ) | 2.97 ( 45.54 ) | 2.96 ( 2.24 ) | 1.57 ( 1.08 ) |
| Flatulence | 31 | 3.32 ( 2.34 - 4.73 ) | 3.32 ( 50.11 ) | 3.31 ( 2.47 ) | 1.73 ( 1.22 ) |
| Covid-19 pneumonia | 30 | 4.72 ( 3.3 - 6.76 ) | 4.71 ( 87.4 ) | 4.7 ( 3.48 ) | 2.23 ( 1.71 ) |
| Limb discomfort | 29 | 4.19 ( 2.91 - 6.03 ) | 4.18 ( 69.99 ) | 4.17 ( 3.07 ) | 2.06 ( 1.53 ) |
| Neuralgia | 26 | 5.19 ( 3.53 - 7.63 ) | 5.18 ( 87.39 ) | 5.16 ( 3.74 ) | 2.37 ( 1.81 ) |
| Cognitive disorder | 26 | 2.86 ( 1.95 - 4.2 ) | 2.86 ( 31.32 ) | 2.85 ( 2.07 ) | 1.51 ( 0.96 ) |
| Pollakiuria | 24 | 3.46 ( 2.32 - 5.17 ) | 3.46 ( 41.86 ) | 3.45 ( 2.47 ) | 1.79 ( 1.21 ) |
| Colitis | 24 | 3.1 ( 2.08 - 4.63 ) | 3.1 ( 34.05 ) | 3.09 ( 2.21 ) | 1.63 ( 1.05 ) |
| Blood pressure abnormal | 19 | 4.45 ( 2.83 - 6.98 ) | 4.44 ( 50.56 ) | 4.43 ( 3.04 ) | 2.15 ( 1.5 ) |
| Urinary incontinence | 19 | 4.01 ( 2.55 - 6.29 ) | 4 ( 42.68 ) | 3.99 ( 2.74 ) | 2 ( 1.35 ) |
| Frequent bowel movements | 19 | 3.39 ( 2.16 - 5.32 ) | 3.38 ( 31.85 ) | 3.38 ( 2.32 ) | 1.76 ( 1.11 ) |
| Pregnancy | 18 | 9.46 ( 5.95 - 15.05 ) | 9.45 ( 135.17 ) | 9.4 ( 6.37 ) | 3.23 ( 2.57 ) |
| Optic neuritis | 18 | 13.27 ( 8.34 - 21.11 ) | 13.25 ( 202.04 ) | 13.14 ( 8.91 ) | 3.72 ( 3.05 ) |
| Transient ischaemic attack | 18 | 4.19 ( 2.64 - 6.66 ) | 4.19 ( 43.57 ) | 4.18 ( 2.84 ) | 2.06 ( 1.4 ) |
| Liver function test increased | 17 | 3.22 ( 2 - 5.18 ) | 3.22 ( 25.9 ) | 3.21 ( 2.16 ) | 1.68 ( 1 ) |
| Bladder disorder | 16 | 8.99 ( 5.5 - 14.71 ) | 8.98 ( 112.82 ) | 8.93 ( 5.92 ) | 3.16 ( 2.46 ) |
| Central nervous system lesion | 16 | 7.98 ( 4.88 - 13.06 ) | 7.98 ( 97.09 ) | 7.94 ( 5.26 ) | 2.99 ( 2.29 ) |
| Muscle spasticity | 15 | 7.58 ( 4.56 - 12.59 ) | 7.57 ( 85.11 ) | 7.54 ( 4.93 ) | 2.91 ( 2.19 ) |
| Bowel movement irregularity | 15 | 8.47 ( 5.09 - 14.07 ) | 8.46 ( 98.07 ) | 8.41 ( 5.5 ) | 3.07 ( 2.35 ) |
| Defaecation urgency | 15 | 10.56 ( 6.36 - 17.56 ) | 10.55 ( 128.79 ) | 10.48 ( 6.85 ) | 3.39 ( 2.67 ) |
| Macular oedema | 14 | 15.19 ( 8.97 - 25.73 ) | 15.18 ( 183.49 ) | 15.03 ( 9.67 ) | 3.91 ( 3.16 ) |
| Cardiac flutter | 13 | 11.66 ( 6.75 - 20.13 ) | 11.65 ( 125.52 ) | 11.56 ( 7.32 ) | 3.53 ( 2.76 ) |
| Chromaturia | 12 | 3.61 ( 2.05 - 6.36 ) | 3.6 ( 22.52 ) | 3.6 ( 2.24 ) | 1.85 ( 1.05 ) |
| Multiple allergies | 12 | 7.95 ( 4.5 - 14.02 ) | 7.94 ( 72.4 ) | 7.9 ( 4.91 ) | 2.98 ( 2.18 ) |
| Peroneal nerve palsy | 11 | 12.75 ( 7.04 - 23.08 ) | 12.74 ( 117.94 ) | 12.63 ( 7.69 ) | 3.66 ( 2.82 ) |
| Hemiparesis | 10 | 4.03 ( 2.17 - 7.51 ) | 4.03 ( 22.74 ) | 4.02 ( 2.39 ) | 2.01 ( 1.14 ) |
| Concussion | 10 | 6.77 ( 3.63 - 12.6 ) | 6.76 ( 48.88 ) | 6.74 ( 4 ) | 2.75 ( 1.88 ) |
| Anal incontinence | 10 | 4.43 ( 2.38 - 8.24 ) | 4.43 ( 26.44 ) | 4.42 ( 2.63 ) | 2.14 ( 1.27 ) |
| Micturition urgency | 10 | 5.25 ( 2.82 - 9.78 ) | 5.25 ( 34.28 ) | 5.23 ( 3.11 ) | 2.39 ( 1.52 ) |
| Magnetic resonance imaging head abnormal | 10 | 38.36 ( 20.47 - 71.9 ) | 38.33 ( 354.18 ) | 37.37 ( 22.09 ) | 5.22 ( 4.34 ) |
| Diarrhoea haemorrhagic | 9 | 5.08 ( 2.64 - 9.78 ) | 5.08 ( 29.4 ) | 5.07 ( 2.93 ) | 2.34 ( 1.43 ) |
| Abnormal faeces | 9 | 5.32 ( 2.76 - 10.24 ) | 5.32 ( 31.43 ) | 5.3 ( 3.07 ) | 2.41 ( 1.49 ) |
| Neoplasm | 9 | 3.74 ( 1.94 - 7.19 ) | 3.74 ( 17.98 ) | 3.73 ( 2.16 ) | 1.9 ( 0.98 ) |
| Lymphocyte count abnormal | 8 | 33.63 ( 16.68 - 67.8 ) | 33.61 ( 247.34 ) | 32.86 ( 18.28 ) | 5.04 ( 4.06 ) |
| Hepatic enzyme abnormal | 8 | 8.03 ( 4.01 - 16.1 ) | 8.03 ( 48.95 ) | 7.99 ( 4.47 ) | 3 ( 2.03 ) |
| Brain fog | 8 | 4.38 ( 2.19 - 8.78 ) | 4.38 ( 20.81 ) | 4.37 ( 2.44 ) | 2.13 ( 1.16 ) |
| Uterine disorder | 7 | 24.81 ( 11.75 - 52.38 ) | 24.79 ( 157.14 ) | 24.39 ( 13.05 ) | 4.61 ( 3.58 ) |
| Vitamin d decreased | 7 | 4.64 ( 2.21 - 9.74 ) | 4.63 ( 19.89 ) | 4.62 ( 2.48 ) | 2.21 ( 1.19 ) |
| Spinal disorder | 7 | 4.11 ( 1.96 - 8.63 ) | 4.11 ( 16.41 ) | 4.1 ( 2.2 ) | 2.03 ( 1.01 ) |
| Extrasystoles | 6 | 6.31 ( 2.83 - 14.07 ) | 6.31 ( 26.67 ) | 6.28 ( 3.21 ) | 2.65 ( 1.56 ) |
| Abdominal tenderness | 6 | 8.27 ( 3.71 - 18.45 ) | 8.27 ( 38.1 ) | 8.22 ( 4.2 ) | 3.04 ( 1.95 ) |
| Trigeminal neuralgia | 6 | 8.87 ( 3.97 - 19.79 ) | 8.87 ( 41.62 ) | 8.82 ( 4.5 ) | 3.14 ( 2.05 ) |
| Gastrointestinal motility disorder | 5 | 5.1 ( 2.12 - 12.27 ) | 5.1 ( 16.4 ) | 5.08 ( 2.44 ) | 2.35 ( 1.16 ) |
| Heart rate abnormal | 5 | 4.2 ( 1.75 - 10.12 ) | 4.2 ( 12.17 ) | 4.19 ( 2.01 ) | 2.07 ( 0.89 ) |
| Bladder pain | 4 | 7.66 ( 2.87 - 20.46 ) | 7.66 ( 23.03 ) | 7.62 ( 3.35 ) | 2.93 ( 1.64 ) |
| Jc polyomavirus test positive | 4 | 9.19 ( 3.44 - 24.57 ) | 9.19 ( 29.01 ) | 9.14 ( 4.01 ) | 3.19 ( 1.9 ) |
| Sleep deficit | 4 | 5.92 ( 2.22 - 15.82 ) | 5.92 ( 16.3 ) | 5.9 ( 2.59 ) | 2.56 ( 1.27 ) |
| Urinary tract disorder | 4 | 5.05 ( 1.89 - 13.47 ) | 5.04 ( 12.92 ) | 5.03 ( 2.21 ) | 2.33 ( 1.04 ) |
| Traumatic fracture | 4 | 24.09 ( 8.97 - 64.73 ) | 24.08 ( 87.05 ) | 23.71 ( 10.37 ) | 4.57 ( 3.26 ) |
| Infrequent bowel movements | 4 | 9.02 ( 3.37 - 24.11 ) | 9.02 ( 28.34 ) | 8.97 ( 3.94 ) | 3.16 ( 1.87 ) |
| Papillary thyroid cancer | 3 | 10.65 ( 3.42 - 33.18 ) | 10.65 ( 26.04 ) | 10.58 ( 4.09 ) | 3.4 ( 1.95 ) |
| Lymph node pain | 3 | 9.45 ( 3.04 - 29.4 ) | 9.44 ( 22.51 ) | 9.39 ( 3.63 ) | 3.23 ( 1.78 ) |
| Hyperaesthesia teeth | 3 | 7.21 ( 2.32 - 22.44 ) | 7.21 ( 15.98 ) | 7.18 ( 2.78 ) | 2.84 ( 1.4 ) |
| Lactose intolerance | 3 | 6.34 ( 2.04 - 19.71 ) | 6.34 ( 13.43 ) | 6.31 ( 2.44 ) | 2.66 ( 1.21 ) |
| Neurological decompensation | 3 | 7.1 ( 2.28 - 22.07 ) | 7.1 ( 15.63 ) | 7.07 ( 2.73 ) | 2.82 ( 1.37 ) |
| Auditory disorder | 3 | 8.4 ( 2.7 - 26.14 ) | 8.4 ( 19.45 ) | 8.36 ( 3.23 ) | 3.06 ( 1.61 ) |
| Cardiac discomfort | 3 | 6.86 ( 2.21 - 21.33 ) | 6.86 ( 14.95 ) | 6.83 ( 2.64 ) | 2.77 ( 1.32 ) |
| Nasal septum deviation | 3 | 9.23 ( 2.96 - 28.71 ) | 9.22 ( 21.86 ) | 9.17 ( 3.55 ) | 3.2 ( 1.75 ) |
| Penile swelling | 3 | 13.64 ( 4.37 - 42.51 ) | 13.63 ( 34.79 ) | 13.51 ( 5.22 ) | 3.76 ( 2.3 ) |
| Clumsiness | 3 | 6.56 ( 2.11 - 20.39 ) | 6.56 ( 14.07 ) | 6.53 ( 2.53 ) | 2.71 ( 1.26 ) |
| Head titubation | 3 | 14.26 ( 4.57 - 44.48 ) | 14.26 ( 36.63 ) | 14.13 ( 5.46 ) | 3.82 ( 2.37 ) |
| Migraine with aura | 3 | 6.23 ( 2 - 19.37 ) | 6.23 ( 13.11 ) | 6.21 ( 2.4 ) | 2.63 ( 1.19 ) |
